# Non-human primate preclinical model revealed the feasibility and short-term safety of iPSC-derived innate-like T cells in autologous transplantation

**DOI:** 10.3389/fimmu.2025.1653275

**Published:** 2026-01-23

**Authors:** Yasuyuki Miyake, Shoichi Iriguchi, Ai Kawana-Tachikawa, Shuichi Kitayama, Eri Imai, Kahoru Taya, Hiroshi Ishii, Tomoyuki Miura, Hiromi Sakawaki, Tetsuro Matano, Shin Kaneko

**Affiliations:** 1 Shin Kaneko Laboratory, Department of Cell Growth and Differentiation, Center for iPS cell research and application (CiRA), Kyoto University, Kyoto, Japan; 2 AIDS Research Center, National Institute of Infectious Diseases, Japan Institute for Health Security, Tokyo, Japan; 3 Department of Latent Infection, National Institute of Infectious Diseases, Japan Institute for Health Security, Tokyo, Japan; 4 Institute for Frontier Life and Medical Sciences, Kyoto University, Kyoto, Japan; 5 National Institute of Infectious Diseases, Japan Institute for Health Security, Tokyo, Japan; 6 Institute of Medical Science, University of Tokyo, Tokyo, Japan

**Keywords:** IPSC, iPSC-derived T cell autologous administration, preclinical, primate model, iPSC-derived

## Abstract

**Background:**

T cells derived from gene-edited induced pluripotent stem cells (iPSCs) are a promising alternative cell source for universal T-cell immunotherapy. However, current preclinical evaluations of iPSC-derived T cells (iPSC-T cells) rely on immunodeficient mouse models, which limit the assessment of immune-related adverse events and in vivo immune cell interactions.

**Methods:**

To overcome these limitations, we developed a preclinical nonhuman primate model to evaluate the safety and cellular kinetics of iPSC-T cells. iPSCs were generated from peripheral blood T cells obtained from two rhesus macaques and redifferentiated in vitro into CD8αβ^-^CD3^+^ innate-like T cells. Phenotypic and functional characterization was performed using flow cytometric analyses. The proliferative capacity of iPSC-T cells was assessed by repeated stimulation with phytohemagglutinin (PHA), and cytotoxic function was evaluated through co-culture assays with target cells. The cells were further transduced with GFP using retroviral vectors during expansion. Autologous GFP⁺ iPSC-T cells were administered to the donor macaques in three separate infusions to assess in vivo safety and cellular kinetics.

**Results:**

The iPSC-T cells exhibited both antigen-dependent and antigen-independent cytotoxicity and demonstrated robust proliferative capacity upon repeated stimulation. Stable GFP expression was maintained during cell expansion. Following autologous infusion, no safety concerns were observed for up to one year after the first administration. Cellular kinetic analyses revealed that the infused iPSC-T cells trafficked to the alveolar space and were no longer detectable in peripheral circulation by seven days post-infusion.

**Conclusion:**

These findings establish a unique immunocompetent primate model for assessing the safety and *in vivo* behavior of iPSC-T cells. This platform enables more physiologically relevant preclinical evaluation and supports the development of iPSC-derived T-cell immunotherapies for cancer and autoimmune diseases.

## 1 Introduction

Immunotherapies including adoptive T-cell transfer and prevention or therapeutic cancer vaccine have long been investigated to treat patients with cancer. Cancer immunotherapies in particular have gained wide attention when immune checkpoint inhibitors and chimeric antigen receptor (CAR)-T cell therapies have demonstrated outstanding clinical responses against solid tumors and B-cell malignancies, respectively (1–4). These achievements called for further development that would improve therapeutic efficacy to other cancers. For example, combination therapy of these new modalities is actively examined for certain cancers (5). In addition, efforts are underway to address issues associated with manufacturing of engineered T cells. These issues include high costs, limited availability, manufacturing time delay, and heterogeneity of products (2, 6). Development of alternative cell sources for engineering T cells would be expected to overcome these obstacles.

T cells differentiated from induced pluripotent stem cells (iPSCs) are a candidate for alternate T-cell sources (7, 8). Several researchers including ours have shown that T cells redifferentiated from iPSCs (iPSC-T) demonstrated therapeutic effects when they were administered into immunodeficient mice carrying tumors (9, 10). Importantly, rejuvenated T cells maintained antigen specificity identical to the original T-cell clone from which they are derived due to the fact that they have the same CDR3 region (11). iPSC-T cells redifferentiated from iPSCs could be distinguished from CD8αβ^+^ and CD8αα^+^. The CD8αα^+^ iPSC-T cell has innate-like phenotype and weaker TCR-dependent cytotoxicity than CD8αβ iPSC-T cell (10, 12). Moreover, the use of CAR propagates target antigen-dependent cytotoxicity (9). The high proliferative ability at both iPSC expansion and iPSC-T cell makes them an ideal platform to generate universal T-cell sources by editing human leukocyte antigens (13–15). Collectively, these studies indicate the feasibility of the production of universal T cells from iPSCs, and they would be an ideal cell source to “off-the-shelf” T-cell immunotherapy. If allogeneic iPSC-T-cell treatments are proved to be safe and effective, T-cell immunotherapy would be applicable to a wide range of patients.

Tumorigenicity, risks of contamination of undifferentiated cells from iPSCs, and unexpected immunogenicity have to be carefully assessed for clinical development. Despite these concerns, all reports used immunodeficient mice to evaluate iPSC-T cell therapeutic effects and safety studies. Although a xenotransplantation model with immunodeficient mice is the only preclinical model of choice to test differentiated cells from human iPSCs, this model cannot fully address these issues. Xenograft transplantation does not replicate the complex relationships of individual cells because of species specificity involving cytokines, ligands, and so on. To more accurately evaluate the safety and the effect of iPSC-T, a non-human primate (NHP) allograft model would be an alternative preclinical model (16).

In the present study, we show the development of an autologous NHP model to test the safety and cellular kinetics of T cells derived from rhesus macaque (rh) iPSCs reprogrammed from T cells. We demonstrate that a total of three-time intravenous administrations of redifferentiated T cells was safe, with no signs of accompanying severe side effects. By retrovirally transducing green fluorescent protein into redifferentiated T cells, we succeed to monitor infused cells in blood over time and find that a fraction of cells migrated into the alveoli of the recipients. Collectively, the present model when coupled with the SIV infection models and applied to allogeneic transplantation models would serve as an important model to propel clinical development of iPSC-derived T cells.

## 2 Results

### 2.1 Generation of iPSCs from rhesus macaque T cells

**Figure 1:**
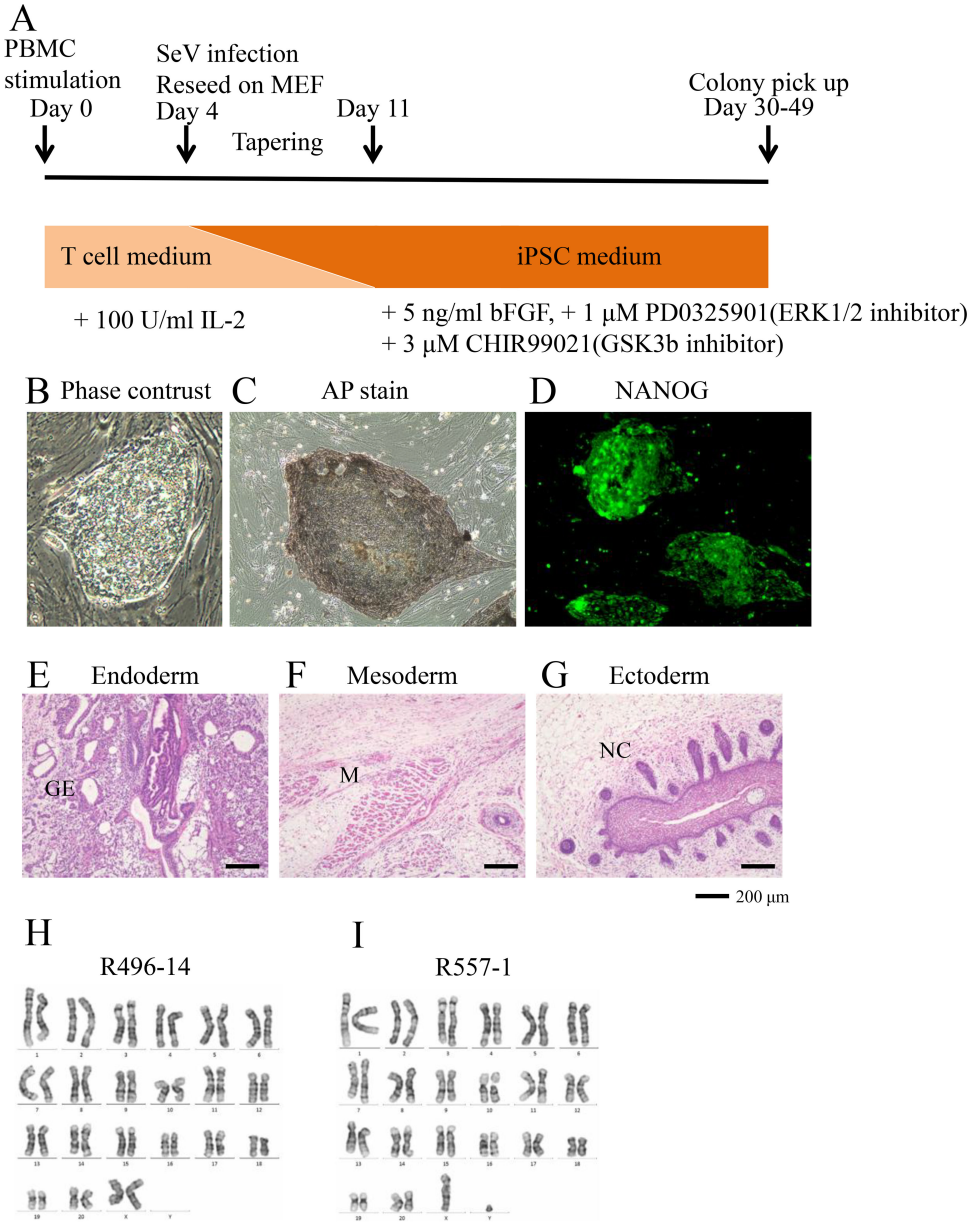
Generation of iPSCs from rhesus monkey peripheral blood T cells **(A)** A schematic showing the derivation of rhT-iPSCs. On day 0, T cells were stimulated by anti-CD2, anti-CD3, and anti-CD28 conjugated beads. Four days after, T cells were infected with SeV expressing yamanaka 4 factors and SV40 large T antigen and were cultured on MEF. In the tapering step, T-cell medium was gradually replaced by 2i medium. iPSC colonies were picked up after day 30. **(B–D)** Characterizations of rhT-iPSC clone R557-1 (as shown in **Table 1**). A phase-contrast image of rhiPSCs **(B)**. A phase-contrast image of iPSCs stained with alkaline phosphatase. *n* = 2 (independent experiments) **(C)**. A fluorescent image of iPSCs stained with an Alexa Fluor 488-conjugated antibody against NANOG. *n* = 2 (independent experiments) **(D–G)** Teratomas derived from rhesus iPSCs were stained by hematoxylin–eosin staining. Teratoma sections show three germ layers: endoderm: GE (gut-like epithelium) **(E)**; mesoderm: M (muscle tissue) **(F)**; and ectoderm: NC (neural crest) **(G)**. Scale bar: 200 μm. **(H, I)** Chromosomal analysis of Rh-iPSCs showing G-banding images of an R496–14 cell **(H)** and an R557–1 cell **(I)**. Six cells were analyzed for each cell line.

We have attempted to generate iPSCs from T lymphocytes in peripheral blood of rhesus monkeys via reprogramming with Sendai virus (SeV) vectors carrying OCT3/4, KLF4, SOX2, c-MYC, LIN28, and SV40 large T antigen as we have previously reported (11, 17). As expected, the combinatorial addition of an ERK1/2 inhibitor, PD0325901, and a GSK3β inhibitor, CHIR99021, during the reprogramming phase resulted in the generation of iPSCs from T cells obtained from independent monkeys (**Figure 1A**). Pluripotency of rhesus T-iPSCs (rhT-iPSCs) was confirmed *in vitro* by their morphology (**Figure 1B**), alkaline phosphatase staining (**Figure 1C**), and immunohistochemical staining for NANOG (**Figure 1D**). Following subcutaneous injection into immunodeficient mice, rhesus T-iPSCs formed a teratoma containing all three germ layers (**Figures 1E, G**). A total of eight SeV transgene-free clones derived from two animals (R496 and R557) were selected for further experiments (**Table 1**). Finally, these Rh-iPSCs were confirmed to be the normal karyotype (**Figures 1H, I**).

**Table 1:** Generation of rhesus T-iPSCs from activated T cells.

Donor ID	Clone name	SeV
R496	1	+
	2	–
	3	+
	7	–
	14	–
R557	1	–
	2	+
	3	–
	4	–
	5	+
	6	–
	8	+
	9	–
	10	+

cDNA derived from the iPSC clone were amplified by Sendai virus vector-specific primers to check the remaining Sendai virus. Out of 14 clones, 6 remained Sendai virus positive.

### 2.2 Redifferentiation of T cells from rhT-iPSCs

**Figure 2:**
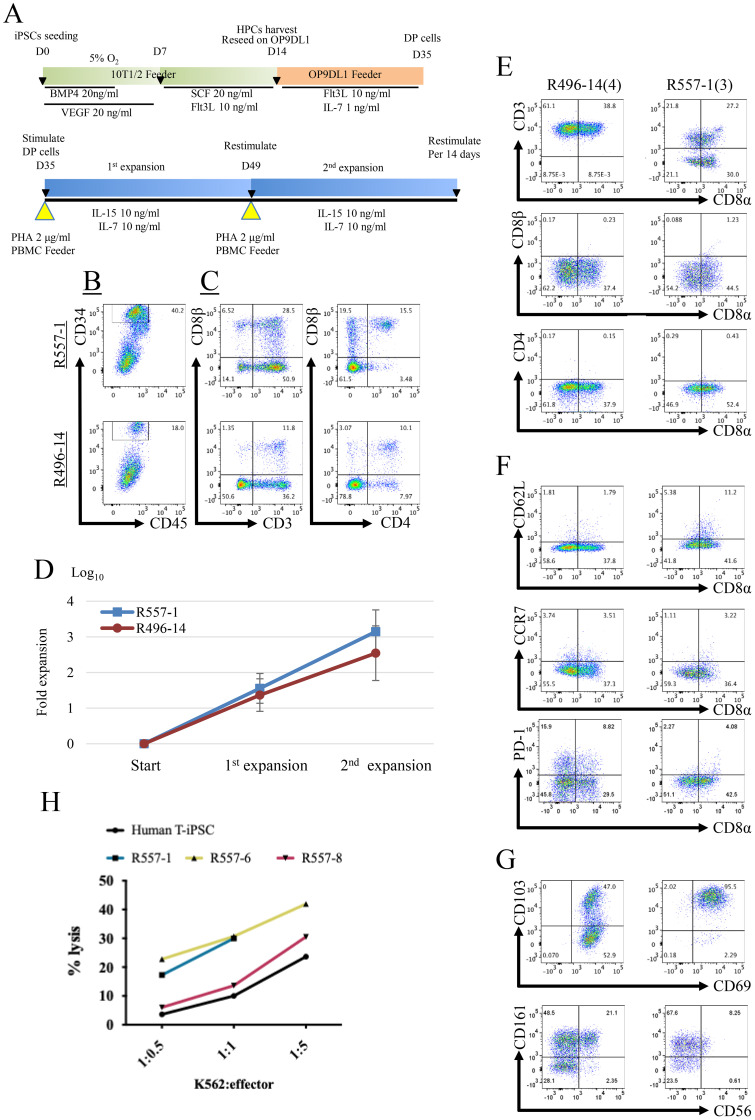
CD8α T-cell differentiation of rhesus iPSCs **(A)** A schematic illustrating the redifferentiation of T cells from iPSCs. iPSC clumps were seeded on C3H10T1/2 Feeder cells. For the first 14 days, iPSCs were differentiated into HSPCs. HSPCs were harvested and reseeded on OP9 feeder cells expressing DL1 to induce differentiation into T cells. Differentiated T cells were cocultured with the human PBMC feeder and 2 μg/mL PHA for TCR stimulation. PHA stimulation was repeated every 2 weeks for T-cell expansion. **(B, C)** Flow cytometric analysis of differentiating cells. The plots show expressions of hematopoietic progenitor cell markers on day 14 **(B)** and expressions of T-cell markers on day 35 **(C)**. CD34 and rhesus CD45 were used as hematopoietic progenitor differentiation markers. CD3, CD4, and CD8β were used as T-cell lineage differentiation markers. **(D)** Kinetics of cell numbers after PHA/PBMC stimulations. The numbers in parentheses indicate total stimulation times. R557–1 *n* = 8 (independent experiments), R496–14 *n* = 4 (independent experiments). Data represent mean ± standard deviations. **(E-G)** Flow cytometric analysis of redifferentiated T cells. Representative flow diagrams showing T-cell phenotype marker expressions on repeatedly stimulated iPSC-T cells **(E)**. The numbers in parentheses indicate total stimulation times. CD3^+^ and CD8a^+^ T lineage cells were sustained after multiple stimulation. **(F)** CD62L and CCR7 were used to detect naïve/memory T cells. PD-1 was used to detect exhausted T cells. **(G)** CD103 and CD69 were used to detect tissue-resident T cells. CD56 and CD161 were used to detect innate like T cells. **(H)** Antigen-independent killing of redifferentiated T cells to the K562 cell line is shown in the line graph. PBMC, peripheral blood mononuclear cells. Plots in **(B**, **C**, **E–G)** are representative of four (R557) or three (R496) independent experiments, respectively.

In order to generate T cells, rhT-iPSCs were induced to differentiate into the hematopoietic lineage by coculturing with a murine cell line, C3H10T1/2, supplemented with VEGF and BMP4 for the first 7 days, and SCF and FLT3L for the next 7 days as shown in **Figure 2A**. On the 14th day after induction, differentiating cells containing CD34^+^CD45^+^ cells were harvested and seeded onto another murine cell line, OP9-DLL1, in the presence of FLT3L and IL-7 to induce T-cell differentiation (**Figure 2B**). Flow cytometric analysis of day 21 after T-cell differentiation demonstrated that majority of the differentiating cells expressed CD3 and approximately 10% of the cells expressed both CD4 and CD8β (**Figure 2C**), indicating that rhT-iPSCs successfully differentiated into T cells with the protocol similar to T-cell differentiation from human iPSCs (11, 18). As summarized in **Table 1**, we observed some interclonal variations in T-cell differentiation efficiency. After differentiating the eight transgene-free iPSCs, we selected R557–1 and R496–14 for further analysis.

Resulting cells from day 21 after T-cell differentiation were activated to induce proliferation by coculturing them with inactivated human peripheral blood mononuclear cells (PBMCs) with added phytohemagglutinin (PHA) (**Figure 2A**). RhiPSC-T cells derived from R496–14 and R557–1 clones proliferated on averages of 59-fold (R496-14) and 38-fold (R557-1), respectively, in 14 days after activation and could be activated again in the next round of activation (**Figure 2D**). At the end of two rounds of activation and proliferation, rhiPSC-T cells from the R496–14 clone expressed CD3, but lost CD4 and CD8β expressions (**Figure 2E**). Nonetheless, nearly half of the rhiPSC-T cells from the R557–1 clones were CD3^−^ at this stage (**Figure 2E**). RhiPSC-T cells from both clones showed a CCR7^−^CD62L^−^ phenotype similar to human effector T cells (**Figure 2F**). In contrast to human iPSC-derived T cells, they expressed high levels of CD103 and CD69 (**Figure 2G**). These findings collectively indicate that regenerated rhiPSC-T cells exhibit an immunophenotype similar to innate-like tissue-resident T cells, which is inconsistent with iPSC-T cells differentiated from human T-iPSCs (11, 12, 19).

### 2.3 *In vitro* functions of regenerated rhiPSC-T cells

To evaluate the antigen-dependent *in vitro* effector functions of rhiPSC-T cells, we transduced them with CD20 CAR and performed cytotoxic assays using PBMCs obtained from autologous monkeys (**Supplementary Figure S1A**). As shown in **Supplementary Figure S1B**, we confirmed that CD20 CAR rhiPSC-T cells recognized and eliminated autologous B cells within PBMCs (**Supplementary Figure S1B**). Cytotoxicity of CD20 CAR rhiPSC-T cells was lower than that of rhesus primary CAR-T (**Supplementary Figure S1C**). Moreover, rhiPSC-T cells killed K562 cells, demonstrating their TCR-independent NK-cell-like cytotoxicity similar to human iPSC-T cells (**Figure 2H**).

### 2.4 Cellular kinetics of regenerated rhiPSC-T cells in autologous recipients

**Figure 3:**
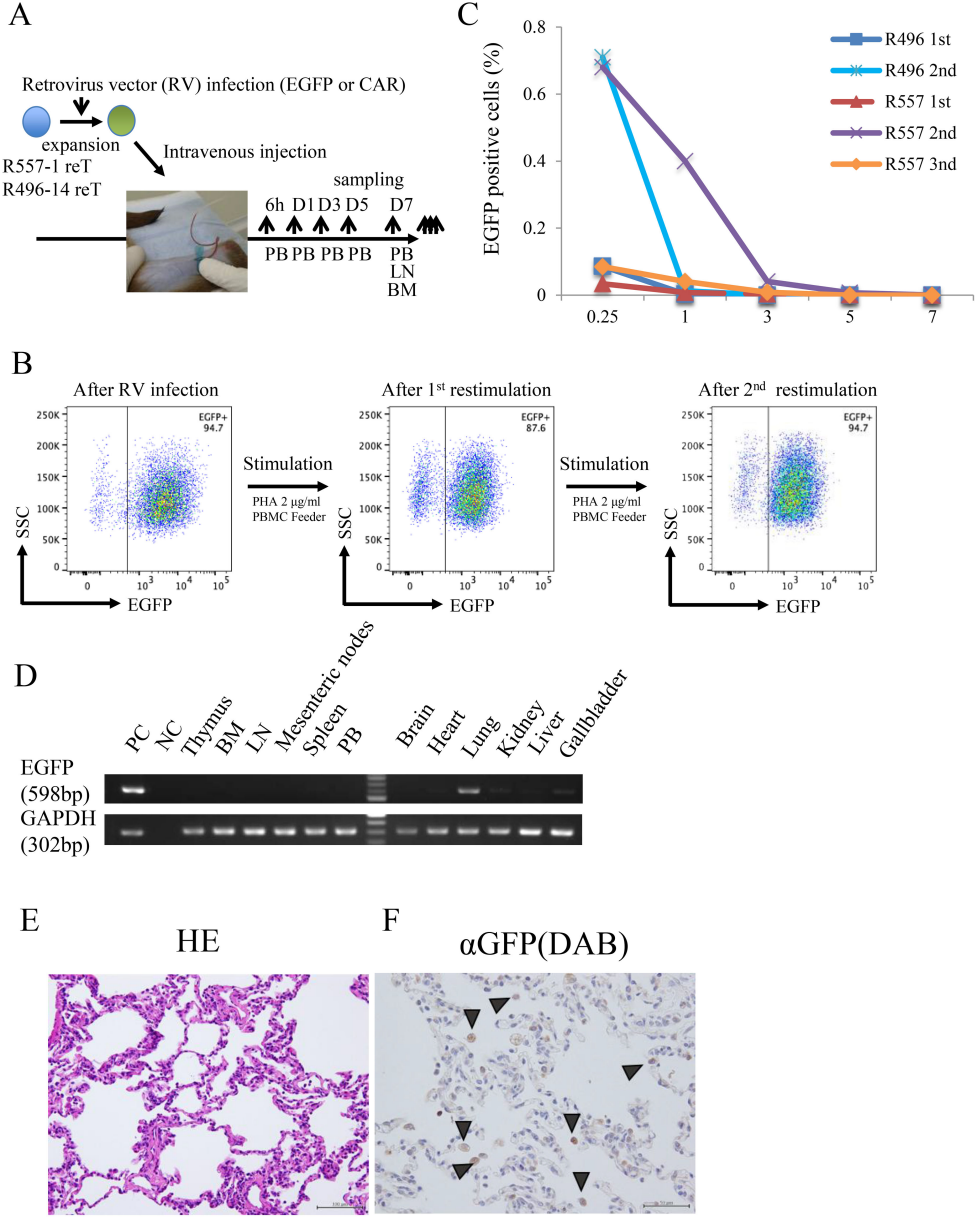
Transplantation of iPSC-T cells **(A)** A schema showing the transplantation and analysis schedule. **(B)** Flow diagrams showing expression of EGFP in iPSC-T cells before and after PHA/PBMC stimulations. **(C)** Cellular kinetics of EGFP^+^ cells in blood samples of recipients at indicated transplantation times measured by flow cytometry. Blue line, first transplantation to R496; dashed light blue, second transplantation to R496; red line, first transplantation to R557; dashed purple line, first transplantation to R557; orange line, third transplantation to R557. R496 tissues were sampled on the second transplantation day. **(D)** Detection of EGPF^+^ cells from peripheral organs of recipient by PCR amplification of the EGFP sequence with specific primers. PC, positive control; NC, negative control; BM, bone marrow; LN, lymph node; PB, peripheral blood cells. gDNA from EGFP-transduced iPSC-T cells was used as the positive control. Distilled water was used as the negative control. GAPDH was used as an internal control of PCR. **(E, F)** Assessment of the infiltration of GFP^+^ cells into the alveoli. Lung sections stained with hematoxylin–eosin **(E)** or immunohistochemically stained with anti-GFP antibody **(F)**. Scale bars, 100 µm **(E)** and 50 µm **(F)**.

Finally, we conducted a series of *in vivo* experiments to understand safety and *in vivo* cellular kinetics of regenerated T cells using the two original monkeys (**Table 2**) (**Figure 3A**). In order to distinguish infused cells from recipient cells, we retrovirally transduced rhiPSC-T cells with EGFP. We evaluated EGFP expressions during *in vitro* culture and found their stable expression (**Figure 3B**). All transplantation studies were carried out with over 8.4 × 10^7^ cells/injection. Approximately 1% of EGFP^+^ cells, which represents infused rhiPSC-derived T cells, were detected in PBMCs at 6 h after transplantation and they subsequently disappeared from the blood by day 7 (**Figure 3C**). Flow cytometric analysis of bone marrow and lymph nodes of the recipients did not show the presence of EGFP^+^ cells (**Supplementary Figure S2**). Given that rhiPSC-T cells expressed tissue-resident markers CD103 and CD69, we examined if rhiPSC-T cells could be detected in non-lymphoid tissues. Semi-quantitative PCR using genomic DNA obtained from R496 monkey tissues showed the presence of EGFP^+^ cells in the lung (**Figure 3D**). To further confirm the presence of iPSC-T cells in the lung, paraffin-embedded lung sections and cytospin samples of bronchoalveolar lavage fluid were prepared and stained with hematoxylin–eosin (HE) and an anti-GFP antibody (**Figures 3E, F**). Microscopic analysis revealed that GFP-positive cells were present in alveolar spaces of the recipients (**Figure 3F**). Transduction of CD20 CAR and preconditioning of recipient R557 prior to infusion did not improve peripheral blood cellular kinetics (**Table 2** and **Figure 3C**). CD20 CAR rhiPSC-T-cell administration did not induce B-cell reduction in blood samples (**Supplementary Figures S3A, B**). These results indicate that rhiPSC-T cells had short *in vivo* persistence and actively migrated to alveoli.

**Table 2:** Transplantation schedules and conditions of autologous iPS-T cells.

Recipient	R557			R496	
Administration number	1st	2nd	3rd	1st	2nd
Transduced gene	EGFP	α20CAR-tEGFR	α20CAR-tEGFR	EGFP	EGFP
			EGFP		
CD3^+^ (%)	49.0	33.6	78.5	99.9	98.8
			80.2		
Infused cell number (10^8^ cells)	1.72	3.78	0.84	1.98	9.93
Pretreatment	–	–	Cyclophosphamide and fludarabine	–	–
Infused date	2016/5/17	2016/9/28	2017/4/18	2016/7/5	2016/11/8
End (observation period)	2016/8/8 (11 weeks)	2016/12/21 (12 weeks)	2017/7/6^*^ (11 weeks)	2016/9/27 (12 weeks)	2016/11/10^*^ (2 days)

Redifferentiated cells were transduced retrovirus vector pDΔN (mock or αCD20-CAR) to distinguish host T cells. Before the second and third transplantation, the absence of residual cell was confirmed by flow cytometric analysis (data not shown). Redifferentiated cells were infused by transvenous administration. Pretreatment was carried out to enhance engraftment. 30 mg/kg cyclophosphamide, 25 mg/m^2^ fludarabine.

*At the end of the observation period, the animals were euthanized.

### 2.5 Safety assessment of autologous infusion

**Figure 4:**
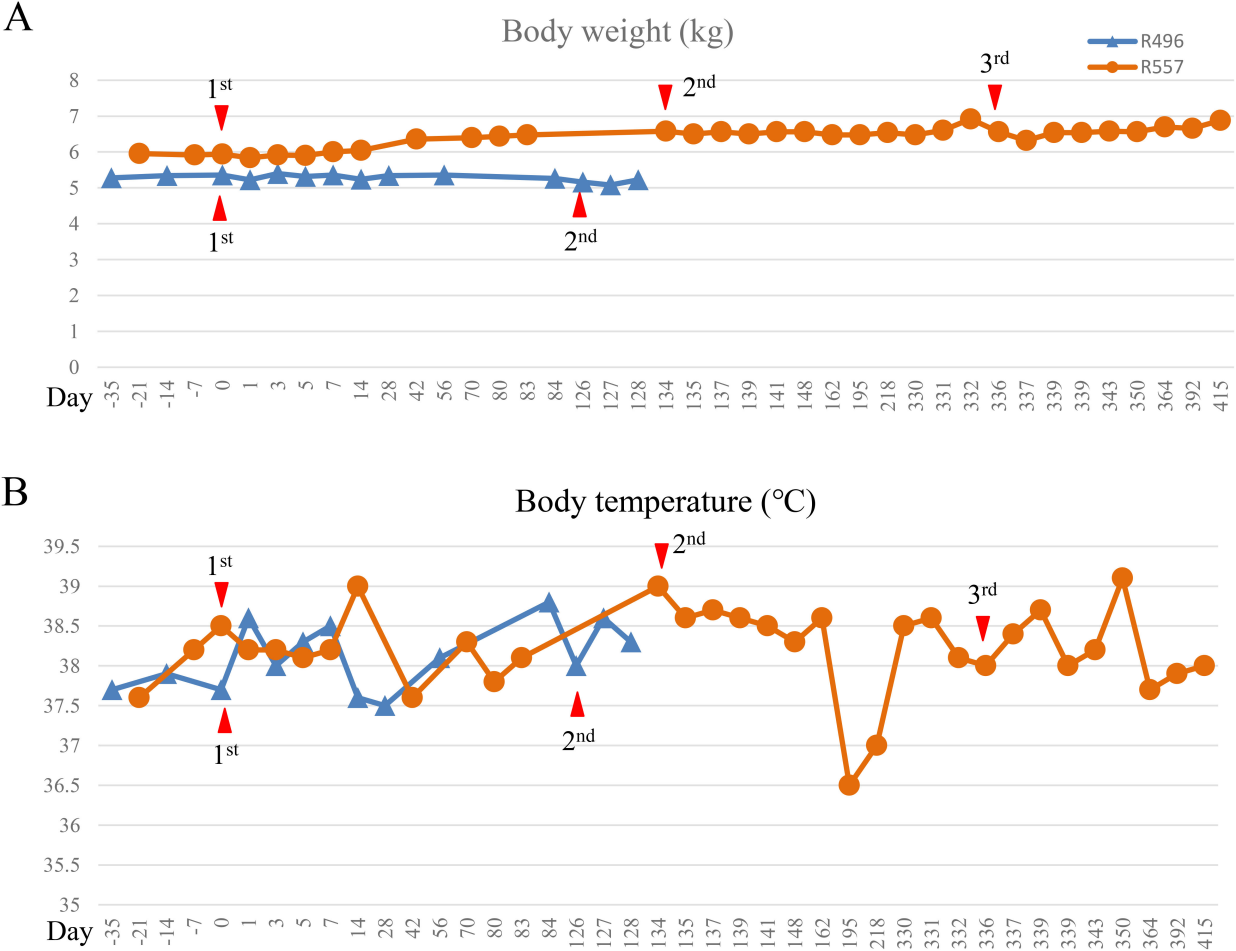
Kinetics of body temperature and body weight of recipient monkeys **(A, B)** Each line graph shows body weight change **(A)** or body temperature change **(B)**. Red line, R557 body weight or body temperature; dashed blue line, R496 body weight or body temperature. Each marker on the line indicates treatment day. Red triangle markers indicate transplantation day.

**Figure 5:**
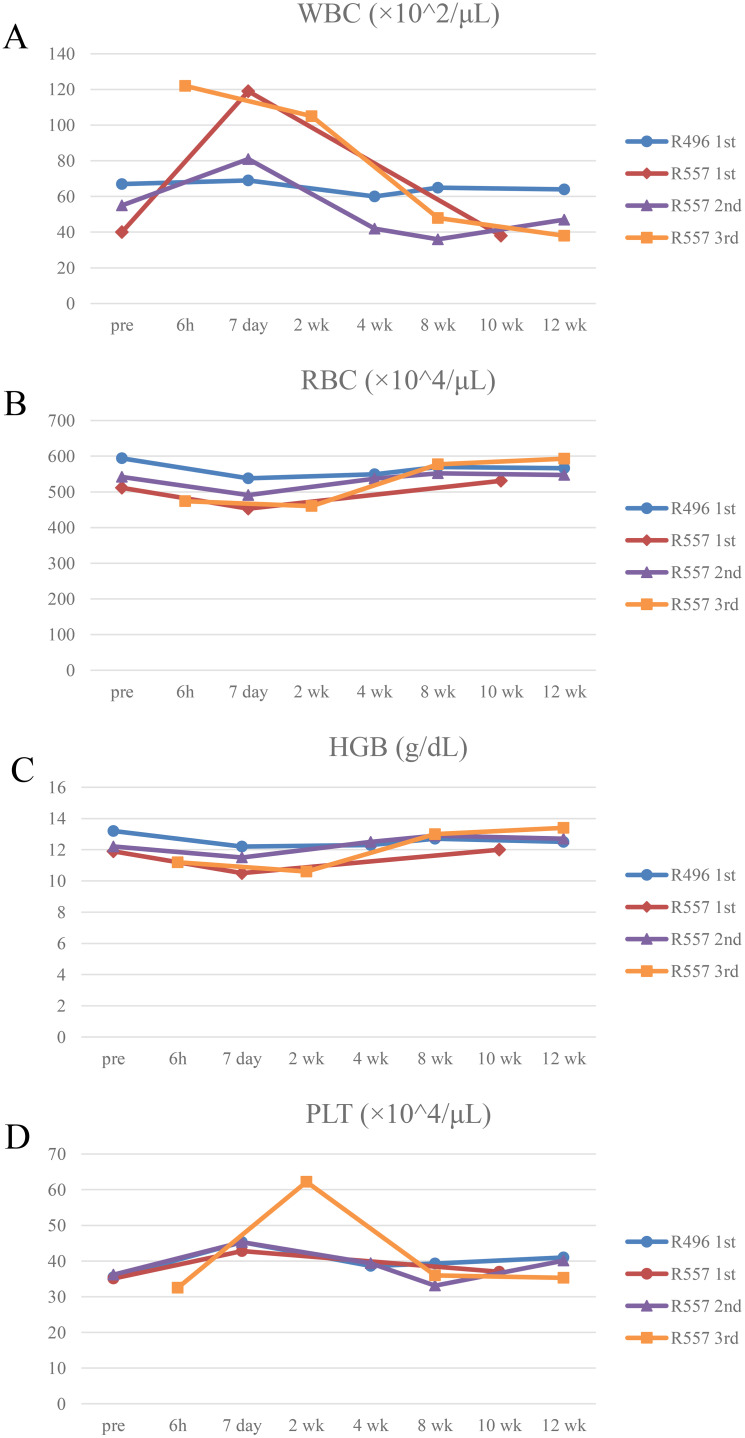
Hematological analysis of recipient monkeys **(A–D)** Complete blood counts measured before and after transplantation. White blood cells (WBC) **(A)**, red blood cells (RBC) **(B)**, hemoglobin (HGB) **(C)**, and platelet (PLT) **(D)**. R496 first transplantation; red line, R557 first transplantation; purple line, R557 second transplantation; orange line, R557 third transplantation.

To assess the side effect of rhiPSC-T cell infusion, we monitored body weight and body temperature (**Figure 4**). Hematological and biochemical analyses were also performed (**Figure 5** and **Supplementary Table S1**). Body weight did not change during the study, except for the third infusion to R557, which included preconditioning (**Figure 4A**). Body temperature remained the normal range of between 39°C and 37.5°C, except in one measurement where the R557 monkey showed transient decrease around day 195 (**Figure 4B**). No remarkable changes in complete blood counts that are categorized to grade III to IV adverse effects were observed. We did not notice notable changes in white blood cell counts, except for the first infusion to R557 (**Figure 5A**). Red blood cell counts and hemoglobin concentrations remained constant (**Figures 5B, C**). A sudden rise in platelet count was observed after 2 weeks of the third infusion to R557 (**Figure 5D**). All changes were transient and recovered by 8 weeks after infusion. Biochemical tests with plasma samples indicated no signs of abnormal elevations or drops in reference to rhesus reference values (20) (**Supplementary Tables S1**–**S4**). Resected organs (brain, lung, heart, thymus, liver, spleen, pancreas, kidney mesentery, and intestine) were histologically assessed for tumorigenesis. Although lymphocyte aggregates that were GFP^−^ were found in hepatic sinusoid, there was no evidence for tumorigenesis in all tissues examined by rhiPSC-T cells (**Supplementary Figures S4A, B**).

## 3 Discussion

Effector immune cells such as T cells and natural killer cells derived from iPS cells may serve as an alternate cell source for allogeneic immunotherapy, but careful safety assessments in preclinical models are a prerequisite to clinical translation. Development of NHP models for iPS cell-derived immune cells will play an important role for this purpose. In this study, we showed the successful differentiation of rhiPSC-T cells from rhesus T-iPSCs and assessment of their cellular kinetics and the safety of autologous rhiPSC-T cell infusions. Importantly, infusions of rhiPSC-T cells three times to monkey did not elicit remarkable safety concerns. In this study, gene transfer was performed using viral vectors, which may lead to random integration of the transgene and potentially cause tumorigenesis. Therefore, an increased copy number of the integrated gene may further elevate the risk of tumorigenesis. Macroscopic and histological analysis of euthanized monkeys showed no signs of tumorigenesis during the observation period in this study. When combined with disease models including the simian immunodeficiency virus infection model or B-cell depletion for the indication of autoimmune diseases, the current model will be an ideal platform to test the therapeutic efficacy and body distribution of TCR- or CAR-engineered rhiPSC-T cells that cannot be evaluated in immunodeficient mouse models.

We have recently demonstrated the generation of rhesus iPSCs from T cells using SeV and redifferentiation into CD34^+^ hematopoietic progenitor cells and macrophages (17). In this report, we showed the successful differentiation of progenitor T cells expressing CD4 and CD8. In addition, rh-iPSC-derived T-cell progenitors were differentiated to innate-like T cells expressing CD8α by a differentiation method similar to human innate-like T-cell generation from human iPSCs (11). Resulting rh-iPSC-T cells proliferated after PBMC/PHA stimulation, but the rate of proliferation was highly variable between experiments. We were able to proliferate rhiPSC-T cells by several rounds of stimulation to harvest a sufficient number of cells for transplantation. The rhiPSC-T cell expansion method allowed us to substantially transduce GFP fluorescent protein for *in vivo* monitoring after autologous infusions and CD20 CAR for antigen-specific *in vitro* assays. In line with our previous studies, those innate-like T cells showed NK cell-like cytotoxicity as it eliminated the K562 cell line *in vitro*.

The first infusion of each monkey was performed to assess the *in vivo* persistence of autologous rhiPSC-T-cell infusion. Few GFP-expressing infused rhiPSC-T cells could be detected in peripheral blood for several days. To examine if short-time persistence resulted in the absence of antigens, we introduced anti-CD20CAR into rhiPSC-T cells to expect their reactivity to autologous B cells, which express the CD20 antigen (21, 22). Recently, clinical indication of CAR-T cells against CD19 antigen has been extended to the treatment of autoimmune diseases such as systemic lupus erythematosus, idiopathic inflammatory myositis and systemic sclerosis (23–25). Although rhiPSC-T cells transduced with anti-CD20 CAR showed cytotoxicity against autologous B cells *in vitro*, they did not show longer persistence and B-cell depletion *in vivo*, suggesting lack of cell-intrinsic properties influencing *in vivo* persistence. It would be possible that T cells differentiated from iPSCs by current differentiation protocols present with innate lymphoid cell-like phenotype and had shorter persistence *in vivo* when compared to adoptive-type T cells. In consistent with these findings, redifferentiated T cells used in this study expressed markers associated with tissue-resident T cells such as CD103 and was detected in the alveoli (19, 26). In mice, infused activated T cells initially travel through pulmonary veins where they infiltrate the pulmonary interstitial compartment (27). The rhiPSC-T cells initially accumulated in the lung using the same mechanism, although rhiPSC-T cells remained in the pulmonary interstitial compartment on day 1 and there were very few cells on day 7. Because of the accumulation of rhiPSC-T cells in the lungs, a temporary slight increase in neutrophil was observed in the alveolar lavage fluid, suggesting that temporary inflammation may have occurred in the lungs (data not shown). When using rhesus iPSCs, the current differentiation method resulted in the appearance of heterogeneous populations such as CD3^−^. In addition, they lack the expression of the beta chain of the CD8 heterodimer, a critical coreceptor for ligating TCR signal to adoptive-type cytotoxic T cells. Previous reports have shown that optimization of positive selection stage culture during T-cell differentiation could lead to the generation of cytotoxic T cells expressing both alpha and beta chains of CD8 (CD8αβ^+^) from human iPSCs (10). Application of these emerging differentiation protocols will improve the *in vivo* persistence of redifferentiated rhiPSC-T cells. In addition to using redifferentiated rhiPSC-T cells with antigen-specific TCR, it is possible to evaluate antigen-specific killing *in vivo* and *in vitro*. We have recently demonstrated that exogeneous cytokine expressions and gene editing improved the persistence and efficacy of T cells differentiated from human iPSCs (15, 28). In particular, the expression of membrane-bound IL-15 and its receptor subunit IL-15Rα may improve the persistence and potency of redifferentiated rhiPSC-T cells (28). Further studies using cytokine-modified rhiPSC-T cells are warranted to investigate the degree of B-cell depletion in this model for assessment for autoimmune diseases associated with B-cell abnormality.

With regard to safety concerns, regular blood tests and daily observations did not show any signs of prolonged adverse events in a total of three infusions over 1 year. Both macroscopic analysis and histological examinations of resected organs at autopsy suggested no abnormal proliferation *in situ* associated with multiple systemic infusions. These findings support the notion that infusion of redifferentiated rhiPSC-T cells used in this study is feasible and did not raise safety concerns. Nonetheless, it would be possible that the short *in vivo* persistence accompanying these T cells may underestimate the safety issues. Moreover, long-term safety assessments remain to be completed.

Finally, in this report, we established an NHP model for rhiPSC-T cells. The NHP model presented here will provide additional insights into the *in vivo* biology of T cells derived from iPSCs and play a critical role for the development of universal T-cell sources against cancer and autoimmune diseases.

## 4 Method

### 4.1 Animal use

Two rhesus macaques (*Macaca mulatta*) were used in this study: one is female (R496, 10 years old) and the other is male (R557, 6 years old). Experiments were carried out at the Institute for Frontier Life and Medical Sciences, Kyoto University after approval by the Committee on the Ethics of Animal Experiments in Kyoto University (permission numbers: B12-1–3 and B12-1-4). Cell transfusion, blood collection, tissue biopsy, and necropsy were performed under ketamine anesthesia. Euthanasia of the rhesus macaques was performed by administering sodium pentobarbital (Kyoritsu Seiyaku) at a dose of 4.212 mg/kg, followed by exsanguination via cardiac puncture.

We used 4- to 8-week-old immunodeficient NOD.Cg-Prkdc^scid^Il2rg^tm1Wjl^/SzJ (NSG). Mice experiments were performed in accordance with the institutional regulations approved by Kyoto University. For euthanasia of mice, CO_2_ was administered at a displacement rate of 30%–70% of the chamber volume per minute.

### 4.2 Rhesus PBMCs

Collected blood was centrifuged at 1,700 rpm for 25 min. The buffy coat was collected and diluted with D-PBS (−) (Nacalai, Japan). Diluted buffy coats were put onto Ficoll-Paque PLUS (GE healthcare) and centrifuged at 540*g* for 25 min. The middle layer was collected as PBMCs.

### 4.3 Inactivation of MEFs

Mouse embryonic feeder cells (MEFs) were seeded 4 × 10^6^ cells on a 10-cm dish in MEF medium [Dulbecco’s modified Eagle’s medium with 10% fetal bovine serum (FBS) and 1% L-glutamine–penicillin–streptomycin (PSG) (Sigma)]. The next day, MEF culture medium was added with mitomycin C (Wako) (fc 10 μg/mL). MEFs were incubated for 90 min at 37°C in a 5% CO_2_ incubator. Medium was removed. Inactivated MEFs were washed with D-PBS (−) three times to remove Mitomycin C. MEF medium was added to MEFs and incubated for 2 h at 37°C in a 5% CO_2_ incubator. MEF medium was removed and MEFs were washed with D-PBS (−). 0.05% Trypsin-EDTA (Sigma) was added to MEFs and incubated for 5 min at 37°C in a CO_2_ incubator. MEF medium was added into MEFs to stop reaction. MEFs were corrected into a centrifugation tube and centrifuged at 1,200 rpm for 5 min. Supernatant was discarded. MEFs were resuspended with DMEM medium. MEFs were seeded at 3 × 10^5^ cells/dish to a 0.1% gelatin (Sigma)-coated six-well plate.

### 4.4 Generation of rhesus T-iPSCs

Rhesus iPSCs were established from T cells as previously described (17). T cells were stimulated by α-NHP-CD3/2/28 antibody-coated beads (Miltenyi Biotec) at a 1:1.5 ratio in R-Medium [RPMI-1640 (Sigma) with 10% FBS and 1% PSG]. Mixture was rotated at approximately 6–10 rpm at room temperature for 45 min. Bead-conjugated cells were separated by a magnet. Separated T cells were suspended with T-cell medium [RPMI-1640 supplemented with 10% FBS, 1% PSG, 0.01 mM 2-mercaptoethanol (Invitrogen), and 100 U/mL recombinant human IL-2 (PeproTech)]. T cells were seeded at 5 × 10^5^ cells/mL in 2 mL of T-cell medium on six-well plates. After 4 days of stimulation, 2 × 10^6^ activated cells were collected in a 15-mL tube and centrifuged at 1,500 rpm for 5 min. Supernatant was removed. T-cell pellets were dissociated by tapping. T-cell suspension was supplemented with 3 MOI of reprogramming factors (Klf4, Oct3/4, Sox4, and c-Myc) and SV40 large T antigen expression SeV vectors. T cells were incubated for 2 h at 37°C. Infected T cells were washed with R-medium and centrifuged at 1,500 rpm for 5 min. Supernatant was removed. Infected cells were suspended in T-cell medium and seeded on inactivated MEFs. Medium was gradually replaced with 2i medium {Dulbecco’s modified Eagle’s medium/F12 (Sigma) supplemented with 20% knockout serum replacement (KSR) (Thermo Fisher Scientific), 1% PSG, 1% nonessential amino acid (Thermo Fisher Scientific), 100 μM 2-mercaptoethanol (Thermo Fisher Scientific), 5 ng/mL basic fibroblast growth factor [bFGF] (Wako; Japan), 1 μM PD0325901 [ERK1/2 inhibitor] (Wako; Japan), and 3μM CHIR99021 [GSK3b inhibitor]} (Tocris). The established iPSC clones were transduced with small interfering RNA L527 (11) and L1913 using Lipofectamine RNAiMAX Reagent (Invitrogen).

### 4.5 Maintenance of Rh-iPSCs

Rh-iPSCs were cultured using a 6-cm dish in 3 mL of 2i medium. Medium was replaced every day and passaging was performed 1 day per week.

### 4.6 Passaging of iPSCs

2i medium was removed from the dish. Rh-iPSCs were incubated with 1 mL/dish dissociation solution [D-PBS (−) (Nacalai; Japan) with 0.25% Trypsin (Invitrogen), 20%KSR, 10 mM CaCl_2_] at 37°C in a 5% CO_2_ incubator for 5 min. After removing the dissociation solution, 1 mL/dish 2i medium was added into the dish. Rh-iPSC clusters were broken up into small cell clumps. Small clumps were seeded onto inactivated MEFs in 2i medium.

### 4.7 Alkaline phosphatase and immunofluorescent stain

Alkaline phosphatase (AP) staining was performed with Alkaline Phosphatase Substrate Kit II (Vector Laboratories, Inc.).

Manufacturer’s protocol was used for AP staining. Stained samples were exposed by a microscope.

Rh-iPSCs were washed with D-PBS (−). Fixative solution was added and incubated at room temperature for 30 s. Fixative solution was removed. Fixed Rh-iPSCs were washed two times with rinse buffer. Staining solution was added and incubated at room temperature for 15 min in the dark. Samples were washed two times with rinse buffer. Samples were filled with D-PBS (−).

### 4.8 Immunofluorescent staining of rhesus iPSCs

To fix the rh-iPSCs, half volume of medium was removed from the iPSC culturing well and the same volume of 4% paraformaldehyde was added. iPSCs were fixed for 30 min at 4°C. Fixing solution was removed. Blocking buffer (0.1% Triton X-100 and 10% Donkey serum) was added and incubated for 30 min at room temperature. Diluted goat anti-NANOG IgG (R&D:AF1997) was added after removing the blocking buffer. Samples were incubated overnight at 4°C. Primary antibody was removed and Donkey anti-goat IgG (H+L) conjugated with Alexa Fluor 488 (Thermo Fisher; A11055) was added. Samples were incubated for 1.5 h at room temperature. After staining, samples were washed with D-PBS (−) and exposed by a fluorescence microscope.

### 4.9 Teratoma assay

2i medium was removed from the dish. One milliliter of 0.05% Trypsin-EDTA was added to the dish and incubated at 37°C for 5 min. Trypsin-EDTA was removed from the dish. D-PBS (−) (1 mL) with 2% FBS was added to the dish. Rh-iPSCs were dissociated by pipetting. Rh-iPSCs were collected into a centrifugation tube and centrifuged at 1,200 rpm for 5 min. Supernatant was removed. iPSCs were suspended with cold D-PBS (−) to 2 × 10^7^ cells/mL. Suspended Rh-iPSCs were mixed with Matrigel in a 1:1 ratio. Cell suspension (200 μL) was injected into 4- to 8-week-old immunodeficient NOD.Cg-Prkdc^scid^Il2rg^tm1Wjl^/SzJ (NSG) subcutaneously under isoflurane anesthesia by a 27G needle-connected syringe. At approximately 2 cm of teratoma diameter or after 11 weeks, teratomas were excised and fixed with neutral buffered formalin at 4°C overnight. The next day, neutral buffered formalin was exchanged with 70% ethanol.

Samples were cut and embedded with paraffin. Paraffin-embedded samples were sectioned and stained by HE staining.

Embedding and HE staining were performed at the Center for Anatomical, Pathological and Forensic Medical Researches (Kyoto University).

### 4.10 Analysis of residual Sendai virus RNA genome

To check residual SeV genome, RNAs were eluted from Rh-iPSCs clones using an RNeasy mini kit (QUIAGEN). cDNAs were synthesized from eluted RNA by a High-Capacity cDNA Reverse Transcription kit (Thermo Fisher Scientific). For amplification of the SeV genome-specific sequence, PCR was performed using primers Fw: 5&-AGACCCTAAGAGGACGAAGA-3& and Rv: 5&-ACTCCCATGGCGTAACTCCATAGTG-3& and ExTaq HS (TAKARA). The PCR condition has three steps (dissociation step: 94°C at 2 min, amplification step: 20 s at 94°C; 40 cycles and elongation step: 5 min at 72°C). The PCR products were electrophoresed in agarose gel.

### 4.11 Karyotype analysis

To arrest Rh-iPSCs at metaphase, Colcemid (Invitrogen) was added to 2i medium at a final concentration of 250 ng/mL. Rh-iPSCs were incubated at 37°C in a 5% CO_2_ incubator for 2 to 3 h. Rh-iPSCs were dissociated with the same method for the passage. The dissociated Rh-iPSCs were collected into a centrifugation tube and centrifuged at 200*g* for 5 min. Supernatant was removed. The iPSCs pellet was suspended with 7 mL of 0.075 M KCl and incubated at room temperature for 20 min. Carnoy’s fixative (7 mL; mixture of methanol and glacial acetic acid in a 3:1 ratio) was slowly added to the cell suspension, followed by gentle mixing. The suspension was centrifuged at 200*g* for 5 min, and the supernatant was removed. The cells were washed twice with 10 mL of Carnoy’s fixative and then centrifuged at 200*g* for 5 min. After the final centrifugation and removal of the supernatant, the cell pellet was resuspended in 5 mL of Carnoy’s fixative and stored at −20°C. Fixed sample was analyzed by researchers at the Tottori Bioscience Promotion Foundation, Japan.

### 4.12 RNA extraction

RNA extractions were performed with an RNeasy mini kit. A total of 1 × 10^6^ cells were harvested from Rh-iPSCs. Rh-iPSCs were suspended with 350 μL of Buffer RLT and the same volume of 70% ethanol was added. Samples were put to the RNeasy mini column. Columns were centrifuged at 10,000 rpm for 15 s. Columns were added with 350 μL of RW1 and centrifuged at 10,000 rpm for 15 s. DNase mixture was applied on columns and incubated for 15 min. Columns were washed with 350 μL of RW1 and centrifuged at 10,000 rpm for 15 s. Columns were added with 500 μL of RPE supplemented with ethanol and centrifuged at 10,000 rpm for 15 s. Columns were added with 500 μL of RPE supplemented with ethanol and centrifuged at 10,000 rpm for 2 min. Collection tube was exchanged for a new one. Columns were centrifuged at 15,000 rpm for 1 min. RNase-free water was added to the column and centrifuged at 10,000 rpm for 1 min. Eluted RNA solutions were stored at −80 °C.

### 4.13 cDNA synthesis

cDNAs were synthesized from eluted RNA by a High-Capacity cDNA reverse Transcription kit. RNA (13.2 μL) was mixed with 2 μL of 10× RT buffer, 0.8 μL of dNTP Mix, 2 μL of RT Random Primers, 1 μL of MultiScribe Reverse Transcriptase, and 1 μL of RNase Inhibitor. Mixed samples were incubated at 25°C for 10 min, at 37°C for 120 min, and at 85°C for 5 min. Samples were stored at −30°C.

### 4.14 Cell lines

DLL1 overexpressing OP9 were cultured in Minimum Essential Medium α (α-MEM) (Invitrogen) supplemented with 15% FBS and 1% PSG. C3H10T1/2 were cultured in Basal Medium Eagle (Invitrogen) supplemented with 10% FBS and 1% PSG. 293GP, MEFs, and FLY-RD18 were cultured in MEF medium. These cells were cultured at 37°C in an atmosphere having a 5% CO_2_ incubator.

### 4.15 T-cell differentiation from iPSCs

To dissociate iPSCs, iPSCs were incubated with dissociation solution at 37 °C in a 5% CO_2_ incubator for 5 min. After removing the dissociation solution and MEFs, the remaining iPSCs clusters were broken up into large cell clumps. iPSC clumps were seeded on C3H10T1/2 feeder cells in HSPC differentiation medium composed of Iscove’s modified Dulbecco’s medium (Sigma) supplemented with 20% FBS, 1% PSG (Sigma), 1% Insulin-Transferrin-Selenium solution (Thermo Fisher Scientific), 450 μM monothioglycerol (Nacalai), 50 μg/mL ascorbic acid 2-phosphate (Nacalai), and 20 ng/mL VEGF (R&D Systems) under 5% O_2_ atmosphere. BMP4 (20 ng/mL; R&D Systems) was added until day 4. After day 7, HSPC differentiation medium was supplemented with 20 ng/mL SCF (R&D Systems) and 10 ng/mL Flt3L (PeproTech). HSPCs were harvested on day 14. HSPCs were seeded on OP9DL1 feeder cells with T-cell differentiation medium [α-MEM with 15% FBS, 1 ng/mL IL7 (PeproTech), and 10 ng/mL Flt3L]. T-cell lineage redifferentiated cells were harvested on day 35.

### 4.16 Expansion of iPSC-T cells

iPSC-T cells were cocultured with irradiated human PBMCs at a 1:20 ratio in expansion medium (RPMI-1640 supplemented with 0.01 mM 2-ME, 10 ng/mL IL-7 and 10 ng/mL IL-15) with 2 μg/mL PHA-P (Wako). The next day, iPSC-T cells were washed two times with D-PBS (−) to exclude PHA-P. iPSC-T cells were maintained with the expansion medium. Half the medium was changed every 2–3 days with re-plating as needed. Stimulation was repeated after the almost 2 weeks of stimulation.

### 4.17 Flow cytometry

All flow cytometry staining was performed with 2% FBS containing D-PBS (−) buffer on ice. PI was added to all samples before analysis. LSR II and FACS Aria II (BD Biosciences) were used for flow cytometry analysis. The following monoclonal antibodies were used for flow cytometry analysis: APC-CD34 (clone 563; BD Biosciences), BV510-rhesus CD45 (clone D058-1283; BD Biosciences), APC-CD3 (clone SP34-2; BD Biosciences), BV421-CD4 (clone OKT4; BioLegend), PerCPcy5.5-CD8α (clone SK1; BioLegend), PEcy7-CD8β (clone SIDI8BEE; eBioscience), PE-CD62L (clone SK11; BD Biosciences), APC-CCR7 (clone G043H7; BioLegend), PEcy7-CD56 (clone HCD56; BioLegend), APC-CD103 (clone 2G5; BECKMAN), APCcy7-CD69 (clone FN50; BioLegend), BV421-CD20 (clone 2H7; BD Biosciences), APC-CD161 (clone HP-3G10; BioLegend), and BV421-PD-1 (clone EH12.2H7; BioLegend). Data were analyzed with FlowJo (Tree Star).

### 4.18 Transfection of retroviral plasmid polyethylenimine (Polysciences Inc.)

293GP cells were seeded on a poly-L-Lysine (Sigma)-coated 10-cm dish. The next day, 3 μg of VSV-G vector and 3 or 6 μg of retrovirus vector were diluted with 500 μL of HBSS (−). Twenty-four microliters of 1 mg/mL polyethylenimine (PEI) (Polysciences Inc.) was added to the vector solution. The solution was mixed vigorously by voltex and incubated for 15 min at room temperature. The mixed solution was added to a 293GP culturing dish and incubated for 16 h in a 5% CO_2_ incubator. After incubation, medium was exchanged with MEF medium with 10 μM Forskolin (Sigma). After 48 h, the supernatant was collected as viral supernatant.

### 4.19 Preparation of retrovirus and infection

At first, VSV-G envelope retrovirus was produced from plasmid-transduced 293GP using PEI. VSV-G retrovirus supernatant was passed through a 0.45-μmφ PVDF syringe filter. FLY-RD18 retrovirus packaging cells were cultured with VSV-G retrovirus supernatant. Retrovirus-infected FLY-RD18 constitutively produce retrovirus having an RD114 envelope (RD virus). Infected FLYRD18 cells were sorted with FACS Aria II. RD virus supernatant was passed through a 0.45-μmφ PVDF syringe filter. RD virus was attached on a retronectin (TAKARA)-coated well. Activated iPSC-T cells were cultured on an RD virus-coated well to infect retrovirus.

### 4.20 Cross-reactivity of CARs

To check the cross-reactivity of anti-human CD19 CAR and anti-human CD20 CAR, each CAR-expressing iPSC-T cell was cocultured with purified rhesus B cells at a ratio of 1:10 in RPMI-1640 supplemented with 10% FBS, 5 ng/mL IL-7, and 20 U/mL IL-2 for 13 h at 37°C in a 5% CO_2_ incubator. Activated rhiPSC-T cells make clusters. Activated iPSC-T cells were exposed by a fluorescence microscope.

### 4.21 Cytotoxic capacity of anti-human CD20CAR to rhesus B cells

PBMCs were centrifuged at 1,200 rpm for 5 min. Supernatant was discarded. PBMCs were incubated for 10 min in HBSS (−) (Nacalai) with 1× CytoTell Red 650 (AAT Bioquest). PBMCs were centrifuged at 1,200 rpm for 5 min and washed with RPMI medium. Stained PBMCs were used to distinguish between rhiPSC-T cells and PBMCs. Anti-CD20 CAR or blank vector (only EGFP) expressing rhiPSC-T cells was harvested after stimulation day 17. iPSC-T cells were washed two times with D-PBS (−) with 2% FBS at 1,200 rpm for 5 min. iPSC-T cells were seeded 1 × 10^5^ cells/well onto a 96-well round-bottom plate. Coculture with stained auto-PBMCs was performed at effector-to-target (E:T) ratios ranging from 1:10 to 1:1 for 24 h at 37°C in a 5% CO_2_ incubator. After coculture, cells were collected and stained with BV421-anti-CD20 antibody and analyzed by FACS Aria II.

### 4.22 Infusion of redifferentiated T cells

Expanded iPSC-T cells were harvested 12 days after stimulation and were passed through a 40-μmφ filter. rhiPSC-T cells were washed two times with D-PBS (−) and resuspended in 20 mL of saline solution containing 2% auto-serum. iPSC-T cells were intravenously administered. Administered rhiPSC-T cell numbers displayed in **Table 2** were calculated as EGFP-positive cells.

### 4.23 Preconditioning regimen treatment

To promote the engraftment of rhiPSC-T cells, we administered the following agents according to the schedule. rhiPSC-T cells were administered on day 0. Cyclophosphamide (30 mg/kg) was administered on day −4. Fludarabine (25 mg/m^2^) was administered on days −6, −5, and −4. Uromitexan (12 mg/kg) was administered two times on day −4. Palonosetron (20 μg/kg) was administered on day −6. Dexamethasone (3.3 mg/body) was administered on days −6, −5, and −4.

### 4.24 Collection of lymphocytes from tissues

#### 4.24.1 Lymph nodes

Excised lymph node was washed with D-PBS (−) with 5 mM EDTA. Lymph node was disrupted and cut as small as possible. Suspension was collected by passing through a 100-μm filter. Corrected suspension was centrifuged at 310*g* for 8 min. Supernatant was discarded and precipitant was suspended with D-PBS (−) containing 5 mM with EDTA. Suspension was put onto Ficoll-Paque PLUS and centrifuged at 1,700 rpm for 25 min. The middle layer was collected and diluted with D-PBS (−) with 2% FBS.

#### 4.24.2 Bone marrow

Collected bone marrow was centrifuged at 1,700*g* for 25 min. The supernatant was discarded. Pellet was diluted with two times volume of D-PBS (−). Diluted bone marrow cells were put onto Ficoll-Paque PLUS and centrifuged at 1,700 rpm for 25 min. The middle layer was collected and diluted with D-PBS (−) with 2% FBS.

#### 4.24.3 Bronchoalveolar lavage fluid

Bronchoalveolar lavage fluid was centrifuged at 1,500 rpm for 5 min and the supernatant was discarded. Cell pellets were suspended with D-PBS (−) with 2% FBS.

### 4.25 Detection of EGFP genomic sequence from organ samples

As shown in **Figure 3C**, organs were isolated from euthanized rhesus at R496 second administration on day 2. Approximately 5-mm square samples were collected from these organs. Samples were incubated in Lysis buffer containing 10 mM Tris-HCl, 0.1 M EDTA, 0.5% (w/v) SDS, and 1 mg/mL Protease K overnight at 55°C. The same volume of phenol–chloroform mixture [Phenol/Chloroform/Isoamyl Alcohol (25:24:1)] was added. Samples were rotated slowly for 10 min. Samples were centrifuged at 15,000 rpm for 5 min, and the upper layer was collected. Sodium acetate (3 M) and ethanol were added. Samples were then rotated slowly for 30 min. Samples were centrifuged at 15,000 rpm for 30 min, and the DNA pellet was washed two times with 70% ethanol solution at 15,000 rpm for 5 min. Dried pellet was resuspended with TE buffer. The concentration of DNA was determined by nanodrop (Thermo Fisher Scientific).

DNA (200 ng) was used for PCR template. For the detection of EGFP, genomic PCR was performed using the primers Fw: 5&-CGAGCTGGACGGCGACGTAAAC-3& and Rv: 5&-GCGCTTCTCGTTGGGGTCTTTG-3& (29) and ExTaq HS (TAKARA). For the detection of GAPDH, genomic PCR was performed using the primers Fw: 5&-TGGAAAAACCTGCCAAGTACG-3& and Rv: 5&-ACCTAGAAGATGAAAAGAGTCGT-3& and ExTaq HS. The PCR condition had three steps (dissociation step: 98°C at 1 min; amplification step 10 s at 98°C, 40 cycles; and elongation step: 5 min at 72°C).

### 4.26 Staining of lung sections

Excluded lung sample was fixed with 4% paraformaldehyde for 42 h at 4°C. Fixation buffer was exchanged with neutral buffered formalin. Fixed sample was analyzed by outsourcing (shinsoshikikagakukenkyujo, Japan).

### 4.27 Hematological and biochemical analysis of blood

Collected blood samples were analyzed by outsourcing (FALCO Biosystems). RBC; Red Blood Cells. WBC; White Blood Cells. HGB; Hemoglobin. HCT; Hematocrit. MCV; Mean Cell volume. MCH; Mean Cell Hemoglobin Concentration. MCHC; Mean Cell Hemoglobin Concentration. PLT; platelet. A/G; albumin/globulin ratio. AST(GOT); asparate aminotransferase. ALT(GPT); alanine aminotransferase. ALP; alkaline phosphatase. LD(LDH); lactate dehydrogenase. γ-GT(γGTP); gamma-glutamyl transpeptidase. CK(CPK); creatine kinase. LDL cholesterol; low density lipoprotein cholesterol. HDL cholesterol; high density lipoprotein cholesterol. CRP; C-reactive protein, quantitative. MCV =Ht (%)/RBC(×10^4^/μL) × 1,000. MCH=HB (g/dL) /RBC(×10^4^/μL) × 1,000. MCHC = Hb(g/dL)/Ht (%) × 100.

## Data Availability

The original contributions presented in the study are included in the article/**Supplementary Material**. Further inquiries can be directed to the corresponding authors.
