# Correction: Non-human primate preclinical model revealed the feasibility and short-term safety of iPSC-derived innate-like T cells in autologous transplantation

**DOI:** 10.3389/fimmu.2026.1883657

**Published:** 2026-05-27

**Authors:** Yasuyuki Miyake, Shoichi Iriguchi, Ai Kawana-Tachikawa, Shuichi Kitayama, Eri Imai, Kahoru Taya, Hiroshi Ishii, Tomoyuki Miura, Hiromi Sakawaki, Tetsuro Matano, Shin Kaneko

**Affiliations:** 1 Shin Kaneko Laboratory, Department of Cell Growth and Differentiation, Center for iPS cell research and application (CiRA), Kyoto University, Kyoto, Japan; 2 AIDS Research Center, National Institute of Infectious Diseases, Japan Institute for Health Security, Tokyo, Japan; 3 Department of Latent Infection, National Institute of Infectious Diseases, Japan Institute for Health Security, Tokyo, Japan; 4 Institute for Frontier Life and Medical Sciences, Kyoto University, Kyoto, Japan; 5 National Institute of Infectious Diseases, Japan Institute for Health Security, Tokyo, Japan; 6 Institute of Medical Science, University of Tokyo, Tokyo, Japan

**Keywords:** IPSC, iPSC-derived T cell autologous administration, preclinical, primate model, iPSC-derived

In the published article, there was a mistake in the **Funding** statement. The **Funding** statement for The Japan Agency for Medical Research and Development in Japan was displayed as “15H04655, JP17fk0410305, JP25fk0410059”. The correct statement is “Japan Agency for Medical Research and Development in Japan (15H04655, JP17fk0410305, JP25fk0410059, JP25fk0410060)’.

The original version of this article has been updated.

