# Supplementary material for: Non-human primate preclinical model revealed the feasibility and short-term safety of iPSC-derived innate-like T cells in autologous transplantation
Source: Front Immunol. 2026 Jan 23;16:1653275. doi: 10.3389/fimmu.2025.1653275 (PMC12875930; doi:10.3389/fimmu.2025.1653275)
Supplement: Supplementary file 1 [file DataSheet1.pdf]

Supplementary Material

Figure S1

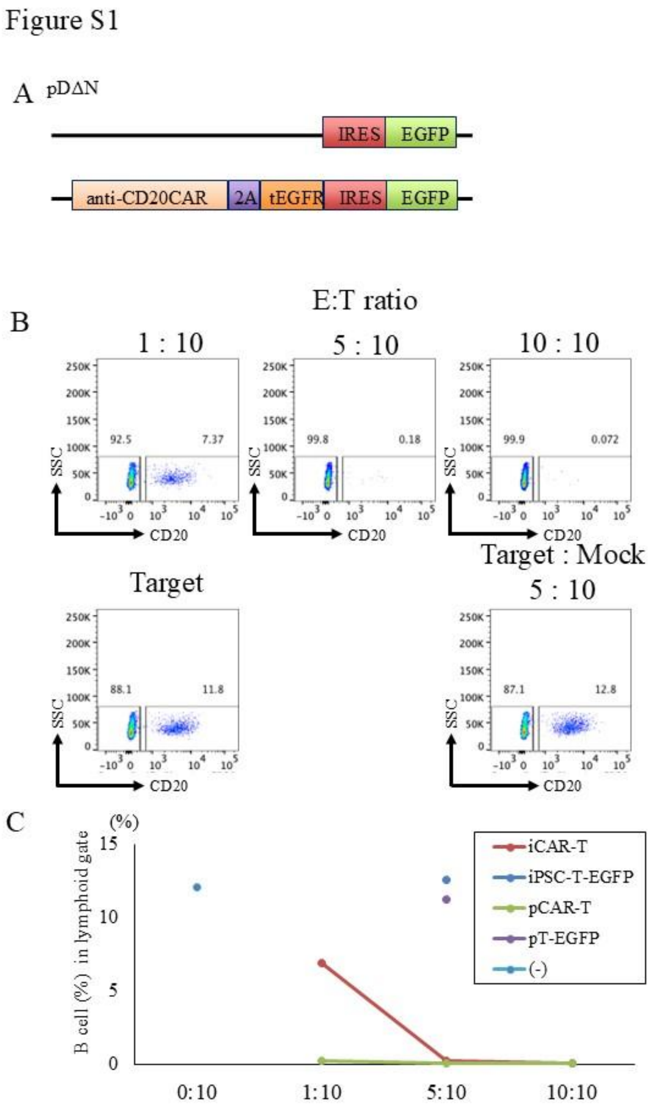

Supplementary Figures 1 Cytotoxicity of αCD20-CAR Expressing iPSC-T cells

(A) pDΔN retrovirus vectors were used for forced expression of transgenes. pDΔN retrovirus vectors contain MSCV promoter and EGFP following IRES element. (B) Flow-cytometric analysis plots showing cytotell650 positive PBMCs. CD20 was used for B cell marker. (Top row) rhesus B cells were eliminated by αCD20-CAR expressing iPSC-T cells in a dose dependent manner. (Bottom left panel) flowdiagram showing culture without effector cells (Bottom right) Flowdiagram showing percentage of $CD20^{+}$ cells when PBMCs were co-cultured with retroviral vector harboring only EGFP (mock). (C) Line graph showing percentages of B cells. red-line, αCD20 CAR-expressing iPSC-T cells (iCAR-T); Blue line, EGFP expressing iPSC-T cell (reT-EGFP); Green line, αCD20-CAR expressing primary T cells (pCAR-T); Purple line, EGFP expressing primary T cells (pT-EGFP); Light blue, only PBMC. n = 2 (technical replicates).

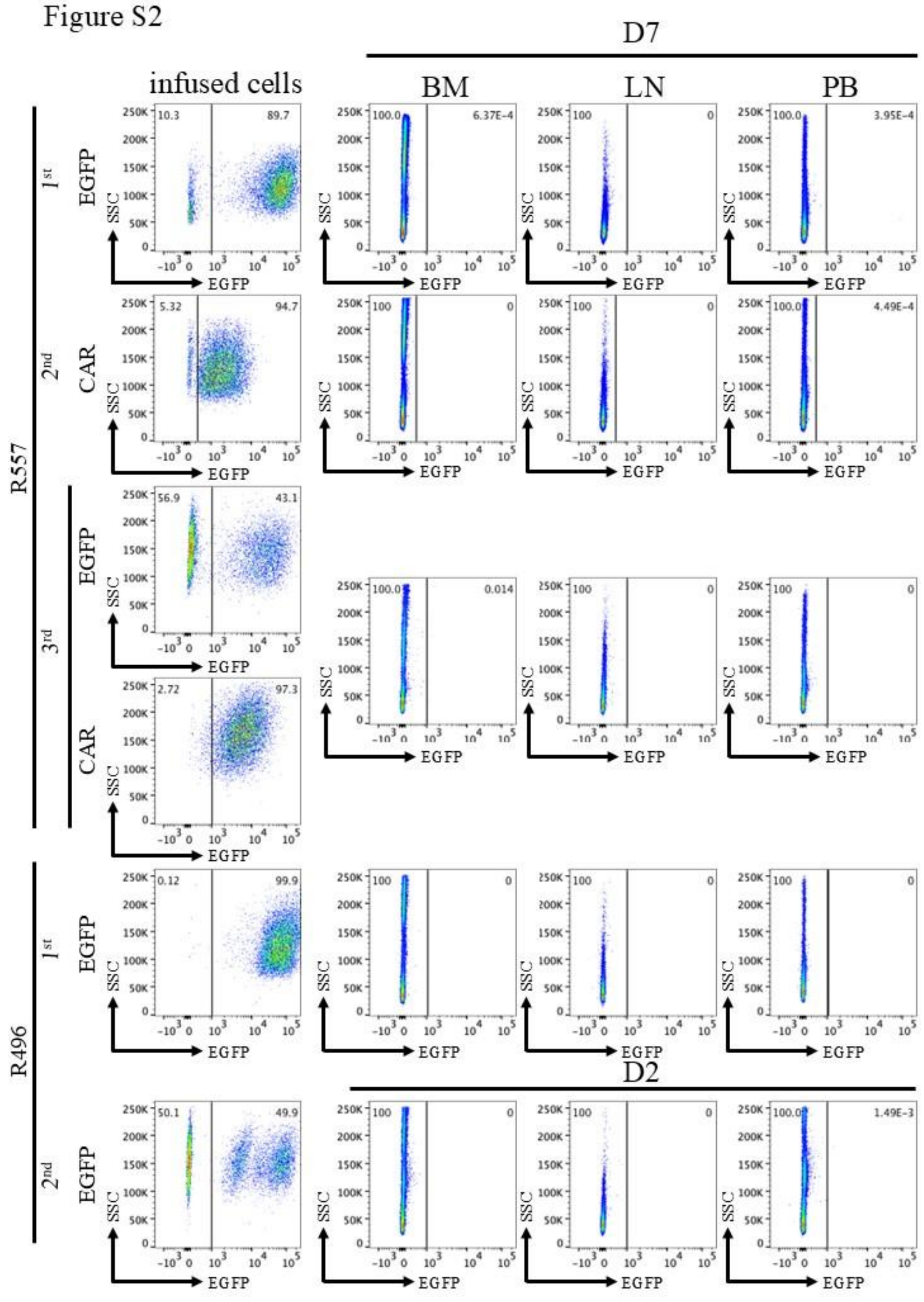

Supplementary Figures 2. Flow-cytometric Analysis of Transplanted Cells in Bone Marrow cells , Lymph Nodes, and Peripheral Blood.

Flowcytometric analysis for detection of infused EGFP+ cells in hematological organs. PBMCs (PB) were isolated from blood samples by Ficoll-paque. Bone marrow (BM) samples were collected from hindlimb and used Ficoll-paque to prepare mononuclear cells. Lymph node (LN) specimen were collected from axillary (1st and 2nd transplantation) and inguinal (3rd transplantation) lymph node. All treatment were performed under anesthesia (R557 1st, 2nd, 3rd and R496 1st transplantation) or after euthanasia (R496 2nd transplantation).

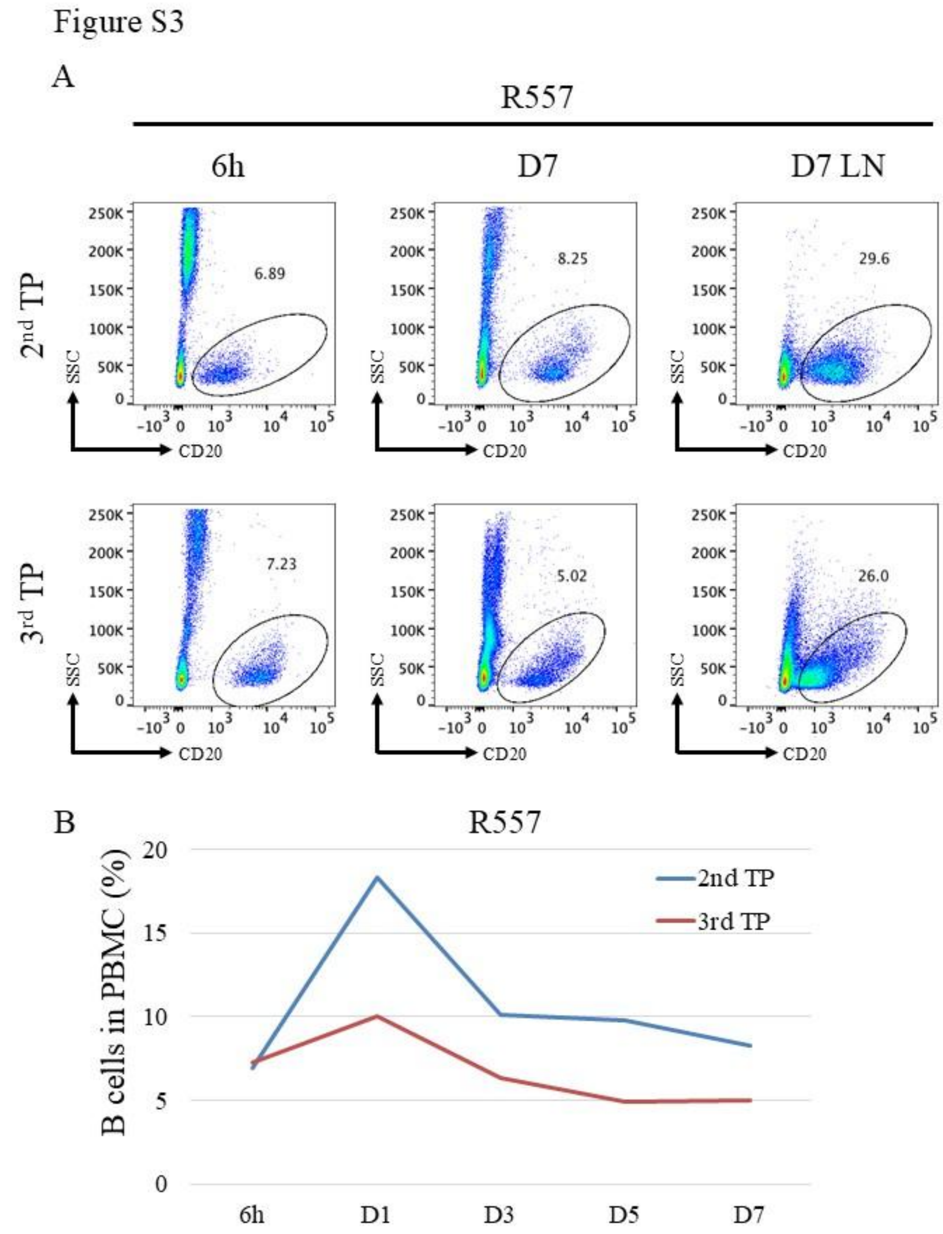

Supplementary Figures 3. Kinetics of Percentage of B cells in PBMCs and Lymph Nodes of the Recipient R557

(A) Flowdiagrams showing percentages of $CD20^+$ cells after 6 hours (Left panels), 7 days (Middle panels) in PBMCs. Flowdiagrams showing percentages of $CD20^+$ cells in lymph node (LN) collected 7 days after infusions are shown (Right panels). (B) Kinetics of B cells after 2nd (blue) and 3rd (red) infusions at indicated hours or days after infusion.

Figure S4

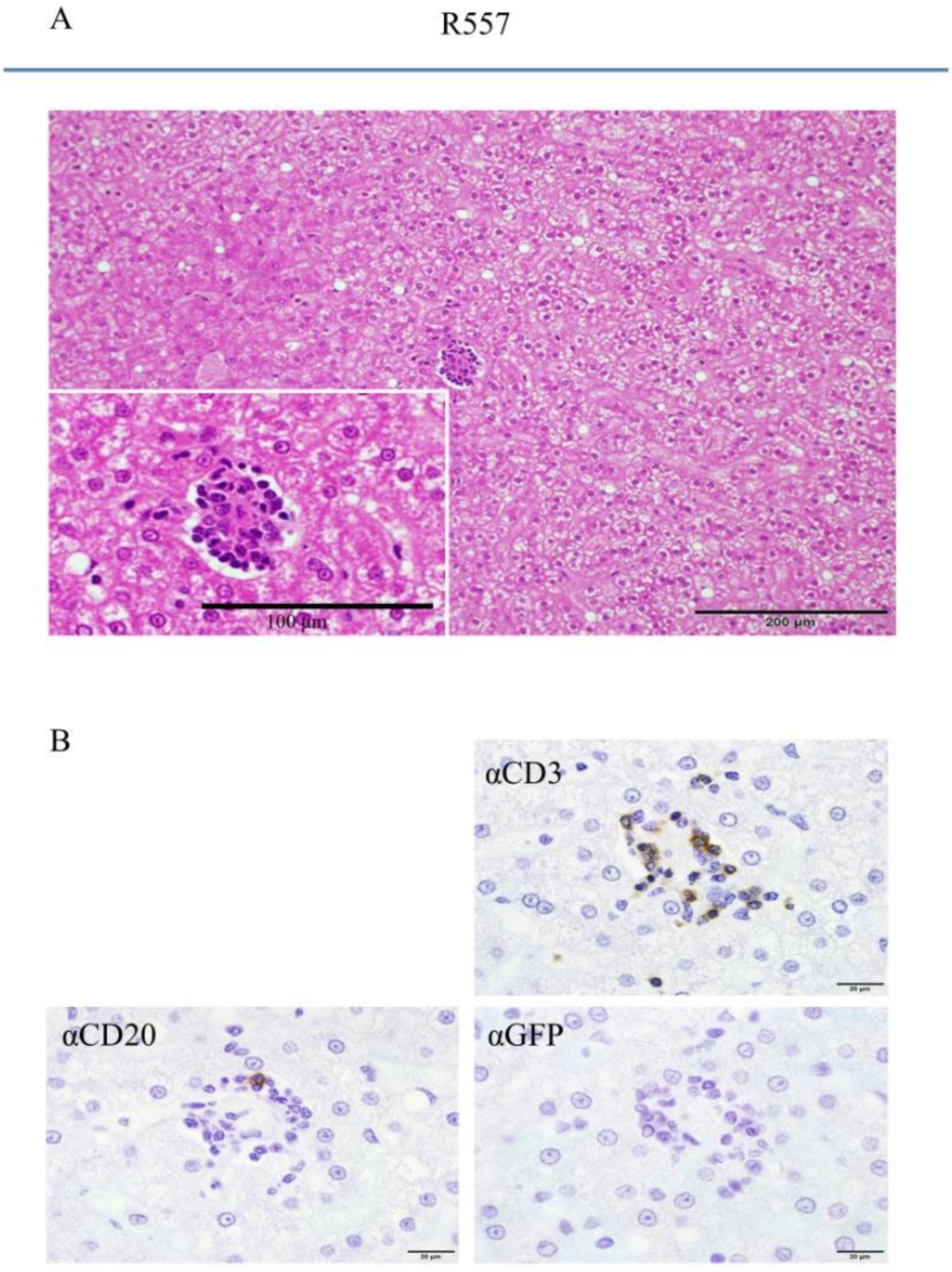

Supplementary Figures 4. Assessment of Tumorigenicity by Histology

(A-B) To assess tumorigenicity, tissue samples (brain, lung, heart, thymus, liver, spleen, pancreas, kidney, mesentery and intestine) were histologically analyzed. An Eosin-Hematoxylin staining show a lymphocyte aggregate in hepatic sinusoid (A). Immunohistochemical analysis of the lymphocyte aggregate stained with anti-CD3 (Top right), -CD20 (Bottom left), and -GFP (Bottom right) antibodies. Scale bar: 200 µm or 100µm (A). Scale bar: 20 µm (B)

Table S1. Hematological and Biochemical analysis of R496 blood following first transplantation

		Pre	7 day post 1st t.p.	4 wk post 1st t.p.	8 wk post 1st t.p.	12 wk post 1st t.p.
RBC	×10^4/μL	594	538	549	570	566
WBC	×10^2/μL	67	69	60	65	64
HGB	g/dL	13.2	12.2	12.3	12.7	12.5
HCT	%	42.3	37.5	39	40.1	39.5
MCV	fL	71.2	69.7	71	70.4	69.8
MCH	pg	22.2	22.7	22.4	22.3	22.1
MCHC	%	31.2	32.5	31.5	31.7	31.6
PLT	×10^4/μL	35.4	45.3	38.6	39.3	41
Basophil	%	0	0	0	0	0
Neutrophil	%	32	22	36	40	57
Eosinophil	%	3	1	1	2	0
Lymphocyte	%	64	76	62	56	40
Monocyte	%	1	1	1	2	3
Total bilirubin	mg/dL	0.1>	0.1>	0.1>	0.1>	0.1>
Direct bilirubin	mg/dL	0.1>	0.1>	0.1>	0.1>	0.1>
Indirect bilirubin	mg/dL	undetectable	undetectable	undetectable	undetectable	undetectable
Total protein	g/dL	6.7	6.8	7.1	6.9	6.8
Alubumin	g/dL	3.8	3.7	3.9	3.9	3.8
A/G		1.3	1.2	1.2	1.3	1.3
AST(GOT)	U/L	30	38	39	32	37
ALT(GPT)	U/L	46	60	54	58	41
ALP	U/L	349	323	315	305	329
LD(LDH)	U/L	200	405	432	344	538
γ-GT(γGTP)	U/L	43	45	51	54	45
Amylase	U/L	290	250	204	216	232
CK(CPK)	U/L	436	350	468	329	307
LDL cholesterol	mg/dL	64	64	73	68	61
Total cholesterol	mg/dL	151	146	164	169	152
Triglycerides	mg/dL	83	85	51	56	45
HDL cholesterol	mg/dL	81	76	93	99	89
Sodium	mEq/L	146	145	144	146	147
Potassium	mEq/L	3.6	3.7	3.6	3.6	3.5
Chloride	mEq/L	107	105	106	109	107
Calcium	mg/dL	8.5	8.8	9	9	8.7
Phosphate	mg/dL	4.7	4.1	5	4.2	4.8
Magnesium	mg/dL	1.8	1.8	1.8	1.9	1.9
Serum iron	μg/dL	146	152	117	110	111
Urea nitrogen	mg/dL	16.5	17.7	16	16.6	17.9
Creatine	mg/dL	0.56	0.58	0.52	0.55	0.63
Urea acid	mg/dL	0.2>	0.2>	0.2>	0.2>	0.2>
Blood glucose	mg/dL	102	128	101	105	92
CRP	mg/dL	0.13	0.19	0.19	0.19	0.15

Fresh heparinized blood samples were used for hematological and biochemical analysis. The left column shows the subject of analysis and the second column shows the unit of each parameter. The upper and the middle lists are complete blood counts and the bottom list shows results of blood chemistry. RBC; Red Blood Cells. WBC; White Blood Cells. HGB; Hemoglobin. HCT; Hematocrit. MCV; Mean Cell volume. MCH; Mean Cell Hemoglobin Concentration. MCHC; Mean Cell Hemoglobin Concentration. PLT; platelet. A/G; albumin/globulin ratio. AST(GOT); Asparate aminotransferase. ALT(GPT); alanine aminotransferase. ALP; alkaline phosphatase. LD(LDH); lactate dehydrogenase. γ-GT(γGTP); gamma-glutamyl transpeptidase. CK(CPK); creatine kinase. LDL cholesterol; low density lipoprotein cholesterol. HDL cholesterol; high density lipoprotein cholesterol. CRP; C-reactive protein, quantitative.

Table S2. Hematological and Biochemical analysis of R557 blood following first transplantation

		Pre	7 day post 1st t.p.	7 wk post 1st t.p.	10 wk post 1st t.p.
RBC	×10^4/μL	511	453	clot formation	531
WBC	×10^2/μL	40	119	clot formation	38
HGB	g/dL	11.9	10.5	clot formation	12
HCT	%	37.6	34.3	clot formation	37.9
MCV	fL	73.6	75.7	clot formation	71.4
MCH	pg	23.3	23.2	clot formation	22.6
MCHC	%	31.6	30.6	clot formation	31.7
PLT	×10^4/μL	35.1	42.8	clot formation	37
Basophil	%	0	0	clot formation	0
Neutrophil	%	43	65	clot formation	42
Eosinophil	%	6	4	clot formation	6
Lymphocyte	%	50	29	clot formation	48
Monocyte	%	1	2	clot formation	4
Total bilirubin	mg/dL	0.1>	0.1>	0.1>	0.1>
Direct bilirubin	mg/dL	0.1>	0.1>	0.1>	0.1>
Indirect bilirubin	mg/dL	undetectable	undetectable	undetectable	undetectable
Total protein	g/dL	6.1	6.8	6.8	6.5
Alubumin	g/dL	4	3.6	3.9	4
A/G		1.9	1.1	1.3	1.6
AST(GOT)	U/L	43	40	57	28
ALT(GPT)	U/L	81	63	125	59
ALP	U/L	508	591	479	512
LD(LDH)	U/L	180	370	151	189
γ-GT(γGTP)	U/L	46	40	42	43
Amylase	U/L	442	471	464	393
CK(CPK)	U/L	309	270	419	277
LDL cholesterol	mg/dL	58	47	47	54
Total cholesterol	mg/dL	115	99	98	106
Triglycerides	mg/dL	43	62	67	42
HDL cholesterol	mg/dL	57	50	48	53
Sodium	mEq/L	147	148	146	147
Potassium	mEq/L	4	4.2	4.4	4
Chloride	mEq/L	109	108	109	110
Calcium	mg/dL	8.7	8.8	8.9	8.9
Phosphate	mg/dL	4.4	3.1	4.2	3.5
Magnesium	mg/dL	1.8	1.7	1.9	1.7
Serum iron	μg/dL	228	116	153	195
Urea nitrogen	mg/dL	21	17.9	22.5	18.6
Creatine	mg/dL	0.68	0.68	0.73	0.77
Urea acid	mg/dL	0.3	0.2>	0.2>	0.2>
Blood glucose	mg/dL	132	118	106	114
CRP	mg/dL	0.07	0.25	0.13	0.09

Table S3. Hematological and Biochemical analysis of R557 blood following second transplantation

		Pre 2nd t.p.	7 day post 2nd t.p.	4 wk post 2nd t.p.	8 wk post 2nd t.p.	12 wk post 2nd t.p.
RBC	×10^4/μL	542	491	537	552	547
WBC	×10^2/μL	55	81	42	36	47
HGB	g/dL	12.2	11.5	12.5	12.9	12.7
HCT	%	38	36.8	41.2	40.9	40
MCV	fL	70.1	74.9	76.7	74.1	73.1
MCH	pg	22.5	23.4	23.3	23.4	23.2
MCHC	%	32.1	31.3	30.3	31.5	31.8
PLT	×10^4/μL	36.2	45.3	39.4	33.1	40.1
Basophil	%	0	1	1	0	0
Neutrophil	%	42	59	41	39	44
Eosinophil	%	5	2	4	2	2
Lymphocyte	%	49	34	48	55	51
Monocyte	%	4	4	6	4	3
Total bilirubin	mg/dL	0.1>	0.1>	0.1>	0.1>	0.1>
Direct bilirubin	mg/dL	0.1>	0.1>	0.1>	0.1>	0.1>
Indirect bilirubin	mg/dL	undetectable	undetectable	undetectable	undetectable	undetectable
Total protein	g/dL	6.7	6.3	6.6	6.5	6.2
Alubumin	g/dL	4.1	3.8	4.1	4.1	4
A/G		1.6	1.5	1.6	1.7	1.8
AST(GOT)	U/L	31	29	20	21	21
ALT(GPT)	U/L	56	55	35	30	33
ALP	U/L	611	452	486	566	528
LD(LDH)	U/L	301	245	148	212	194
γ-GT(γGTP)	U/L	42	38	43	41	40
Amylase	U/L	517	501	407	464	452
CK(CPK)	U/L	218	150	229	297	201
LDL cholesterol	mg/dL	44	39	49	52	54
Total cholesterol	mg/dL	101	86	104	108	114
Triglycerides	mg/dL	49	69	45	45	70
HDL cholesterol	mg/dL	57	44	54	60	56
Sodium	mEq/L	147	148	147	146	146
Potassium	mEq/L	3.9	4.1	4.1	4.7	4.6
Chloride	mEq/L	108	108	109	108	107
Calcium	mg/dL	8.9	8.6	9.1	9	9
Phosphate	mg/dL	4.1	3.3	4	4.7	4.4
Magnesium	mg/dL	1.8	1.9	1.7	1.9	1.9
Serum iron	μg/dL	147	143	139	186	184
Urea nitrogen	mg/dL	19.7	15.1	16	20.5	20.6
Creatine	mg/dL	0.73	0.75	0.8	0.76	0.74
Urea acid	mg/dL	0.2>	0.2>	0.2>	0.2>	0.2>
Blood glucose	mg/dL	121	101	112	135	126
CRP	mg/dL	0.46	0.11	0.06	0.06	0.07

Table S4. Hematological and Biochemical analysis of R557 blood following third transplantation

		6 h post 3rd t.p.	2 wk post 3rd t.p.	8 wk post 3rd t.p.	12 wk post 3rd t.p.
RBC	×10^4/μL	474	460	577	593
WBC	×10^2/μL	122	105	48	38
HGB	g/dL	11.2	10.6	13	13.4
HCT	%	34.8	33.8	41.2	42.8
MCV	fL	73.4	73.5	71.4	72.2
MCH	pg	23.6	23	22.5	22.6
MCHC	%	32.2	31.4	31.6	31.3
PLT	×10^4/μL	32.5	62.2	36	35.3
Basophil	%	0	0	0	0
Neutrophil	%	76	63	46	46
Eosinophil	%	0	1	4	2
Lymphocyte	%	22	34	45	52
Monocyte	%	2	2	5	0
Total bilirubin	mg/dL	0.1>	0.1>	0.1>	0.1>
Direct bilirubin	mg/dL	0.1>	0.1>	0.1>	0.1>
Indirect bilirubin	mg/dL	undetectable	undetectable	undetectable	undetectable
Total protein	g/dL	5.6	6.6	6.8	7
Alubumin	g/dL	3.4	3	4.2	4.3
A/G		1.5	0.8	1	1.6
AST(GOT)	U/L	26	23	22	24
ALT(GPT)	U/L	67	36	43	41
ALP	U/L	520	648	445	600
LD(LDH)	U/L	261	274	121	211
γ-GT(γGTP)	U/L	37	36	44	45
Amylase	U/L	386	420	425	421
CK(CPK)	U/L	234	122	152	253
LDL cholesterol	mg/dL	32	44	50	57
Total cholesterol	mg/dL	89	98	106	113
Triglycerides	mg/dL	57	67	61	45
HDL cholesterol	mg/dL	54	51	56	60
Sodium	mEq/L	148	146	148	148
Potassium	mEq/L	4.3	4.3	4.2	4.2
Chloride	mEq/L	109	108	110	106
Calcium	mg/dL	8.8	9	9.1	9.4
Phosphate	mg/dL	3.4	3.6	4.1	2.6
Magnesium	mg/dL	1.8	1.6	1.8	2
Serum iron	μg/dL	116	126	156	147
Urea nitrogen	mg/dL	13.8	16.6	19.3	17.9
Creatine	mg/dL	0.61	0.65	0.7	0.76
Urea acid	mg/dL	0.2>	0.2>	0.2>	0.2>
Blood glucose	mg/dL	150	90	131	192
CRP	mg/dL	3.77	0.94	0.04	0.06
